# Supplementary material for: Baseline oxygen consumption decreases with cortical depth
Source: PLoS Biol. 2022 Oct 27;20(10):e3001440. doi: 10.1371/journal.pbio.3001440 (PMC9642908; doi:10.1371/journal.pbio.3001440)
Supplement: S1 Appendix — (DOCX) [file pbio.3001440.s001.docx]

Derivation of the ODACITI model

We consider an axially symmetrical tissue configuration with uniform tissue consumption equal ${CMRO}_{2}$. A diving arteriole with radius $R_{ves}$ is positioned along the axis of the tissue cylinder. The periarteriolar space is devoid of the capillaries up to the radius $R_{t}$. Therefore, we assume that inside the cylinder with the radius $R_{t}$, there are no other sources of oxygen except the arteriole. Outside the cylinder with the radius $R_{t}$, we assume that tissue is uniformly filled with the capillaries and that this uniformly distributed oxygen source has a strength equal ${CMRO}_{2}$, perfectly matching tissue consumption. We also consider that ${pO}_{2}=const$ for $r\leq R_{ves}$, that ${pO}_{2}$ is continuous function at $R_{ves}$ and $R_{t}$, and that first derivative of ${pO}_{2}$ is continuous function at $R_{t}$. These assumptions lead to the following set of equations:

:

:

which general solutions can be expressed as:

$R_{ves}\leq r\leq R_{t}$:

$r>R_{t}$:

The substitution of the boundary conditions can be performed in the following way:

1. ${pO}_{2}\left( r\leq R_{ves} \right)={pO}_{2,ves}$ implies

$${pO}_{2,ves}=c_{1}+c_{2}ln(R_{ves})+\frac{{CMRO}_{2}}{4\alpha D}R_{ves}^{2}$$

2. Continuity of ${pO}_{2}$ at $r=R_{t}$ implies

$$c_{1}+c_{2}\ln\left( R_{t} \right)+\frac{{CMRO}_{2}}{4\alpha D}R_{t}^{2}=d_{1}+d_{2}ln(R_{t})$$

3. Continuity of the first derivative of ${pO}_{2}$ at $r=R_{t}$ implies

$$\frac{c_{2}}{R_{t}}+\frac{{CMRO}_{2}}{2\alpha D}R_{t}=\frac{d_{2}}{R_{t}}$$

We further note that when $d_{2}=0$, our model reduces to a well-known Krogh cylinder model. However, for a general case where $d_{2}>0$ (please note that $d_{2}<0$ leads to a non-physiological solution with negative ${pO}_{2}$ values), there is a radius $r=R_{0}$, $R_{0}<R_{t}$, for which oxygen flux through the cylindrical boundary ${r=R}_{0}$ is zero. In this case, the tissue region between $R_{0}$ and $R_{t}$ is supplied with oxygen by the capillary bed, while the tissue region inside $R_{0}$ is supplied with oxygen by the central arteriole. We can use this zero-flux boundary condition at $r=R_{0}$:

$$\frac{c_{2}}{R_{0}}+\frac{{CMRO}_{2}}{2\alpha D}R_{0}=0$$

to express the value of the coefficient $c_{2}$ as a function of another free parameter ($R_{0}$), which has more clear physiological meaning.

Based on the above equations, we can find unique expressions for c1, c2, d1, and d2 and express ${pO}_{2}$ as:

$$r<R_{ves}: pO_{2}\left( r \right)={pO}_{2,ves}$$

$$R_{ves}\leq r\leq R_{t}: pO_{2}\left( r \right)={pO}_{2,ves}+\frac{{CMRO}_{2}}{4D\alpha}\left( r^{2}-R_{ves}^{2} \right)-\frac{{CMRO}_{2}}{2D\alpha}R_{0}^{2}ln\frac{r}{R_{ves}}$$

$$r>R_{t}: pO_{2}\left( r \right)={pO}_{2,ves}+\frac{{CMRO}_{2}}{4D\alpha}\left( R_{t}^{2}-R_{ves}^{2} \right)-\frac{{CMRO}_{2}}{2D\alpha}\left( R_{0}^{2}ln\frac{r}{R_{ves}}-R_{t}^{2}\ln\frac{r}{R_{t}} \right)$$

Fitting for CMRO2

To facilitate the fitting for ${CMRO}_{2}$, we first make the following substitutions:

$$\beta=\frac{{CMRO}_{2}}{2D\alpha}\left( R_{ves}^{2}-R_{0}^{2} \right)$$

$$M_{0}=\frac{CMRO_{2}}{\alpha D}$$

and arrive with the following set of equations:

$$r<R_{ves}: pO_{2}\left( r \right)={pO}_{2,ves}$$

$$R_{ves}\leq r\leq R_{t}: pO_{2}\left( r \right)={pO}_{2,ves}+\frac{1}{4}M_{0}\left( r^{2}-R_{ves}^{2}-2R_{ves}^{2}ln\frac{r}{R_{ves}} \right)+ \beta ln\frac{r}{R_{ves}}$$

$$r>R_{t}: pO_{2}\left( r \right)={pO}_{2,ves}+\frac{1}{4}M_{0}\left( R_{t}^{2}-R_{ves}^{2}+2R_{t}^{2}\ln\frac{r}{R_{t}}-{2R}_{ves}^{2}ln\frac{r}{R_{ves}} \right)+ \beta ln\frac{r}{R_{ves}}$$

Let’s assume that we observed ${pO}_{2}$ at *N* locations in the periarteriolar space (${pO}_{2,obs}\left( r_{i} \right)|_{i=1..N})$. Based on the ODACITI equations, we can write

$$\hat{pO}_{2,obs}=\hat{X}\cdot\hat{\varepsilon}$$

where $\hat{pO}_{2,obs}$ is a column vector with the pO2 measurements

$$\hat{pO}_{2,obs}=\left[ \begin{matrix} {pO}_{2,obs}\left( r_{1} \right) \\ \ldots\\ {pO}_{2,obs}\left( r_{N} \right) \end{matrix} \right]$$

$\hat{\varepsilon}$ is given by

$$\hat{\varepsilon}=\left[ \begin{matrix} M_{0} \\ {pO2}_{ves} \\ \beta\end{matrix} \right]$$

and $\hat{X}$ is expressed as

$$\hat{X}=\left[ \begin{matrix} y_{tmp}(r_{1}) & 1 & y_{logr}(r_{1}) \\ \ldots& \ldots& \ldots\\ y_{tmp}(r_{N}) & 1 & y_{logr}(r_{N}) \end{matrix} \right]$$

Functions inside the matrix $\hat{X}$ are defined in the following way:

$${r<R_{ves}: y}_{tmp}(r)=0$$

$$R_{ves}<r<R_{t}: y_{tmp}\left( r \right) =\frac{1}{4}\left( r^{2}-{R_{ves}}^{2}-2{R_{ves}}^{2}\ln\frac{r}{R_{ves}} \right)$$

$$r>R_{t}: y_{tmp}\left( r \right)=\frac{1}{4}\left( {R_{t}}^{2}-{R_{ves}}^{2}-2{R_{ves}}^{2}\ln\frac{r}{R_{ves}}+2{R_{t}}^{2}\ln\frac{r}{R_{t}} \right)$$

$$r<R_{ves}: y_{log}\left( r \right) =0$$

$${r\geq R_{ves}: y}_{log}\left( r \right)=ln\left( \frac{r}{R_{ves}} \right)$$

When $R_{ves}$ and $R_{t}$ were provided by the measurements, a fitting for $\hat{\varepsilon}$ was performed by simply calculating the following expression in Matlab

$$\hat{\varepsilon}=\hat{X}^{-1}\cdot\hat{pO}_{2,obs}$$

When $R_{ves}$ and $R_{t}$ were also fitted parameters together with the $\hat{\varepsilon}$, we performed fitting by minimizing a cost function in Matlab using a function *fminsearch*. The cost function first calculates $\hat{\varepsilon}=\hat{X}^{-1}\cdot\hat{pO}_{2,obs}$ based on initially assumed values for $R_{ves}$ and $R_{t}$, and then computes a sum of the squares of the differences between predicted and measured ${pO}_{2}$ values.
