# Supplementary figures and images for: Baseline oxygen consumption decreases with cortical depth
Source: PLoS Biol. 2022 Oct 27;20(10):e3001440. doi: 10.1371/journal.pbio.3001440 (PMC9642908; doi:10.1371/journal.pbio.3001440)

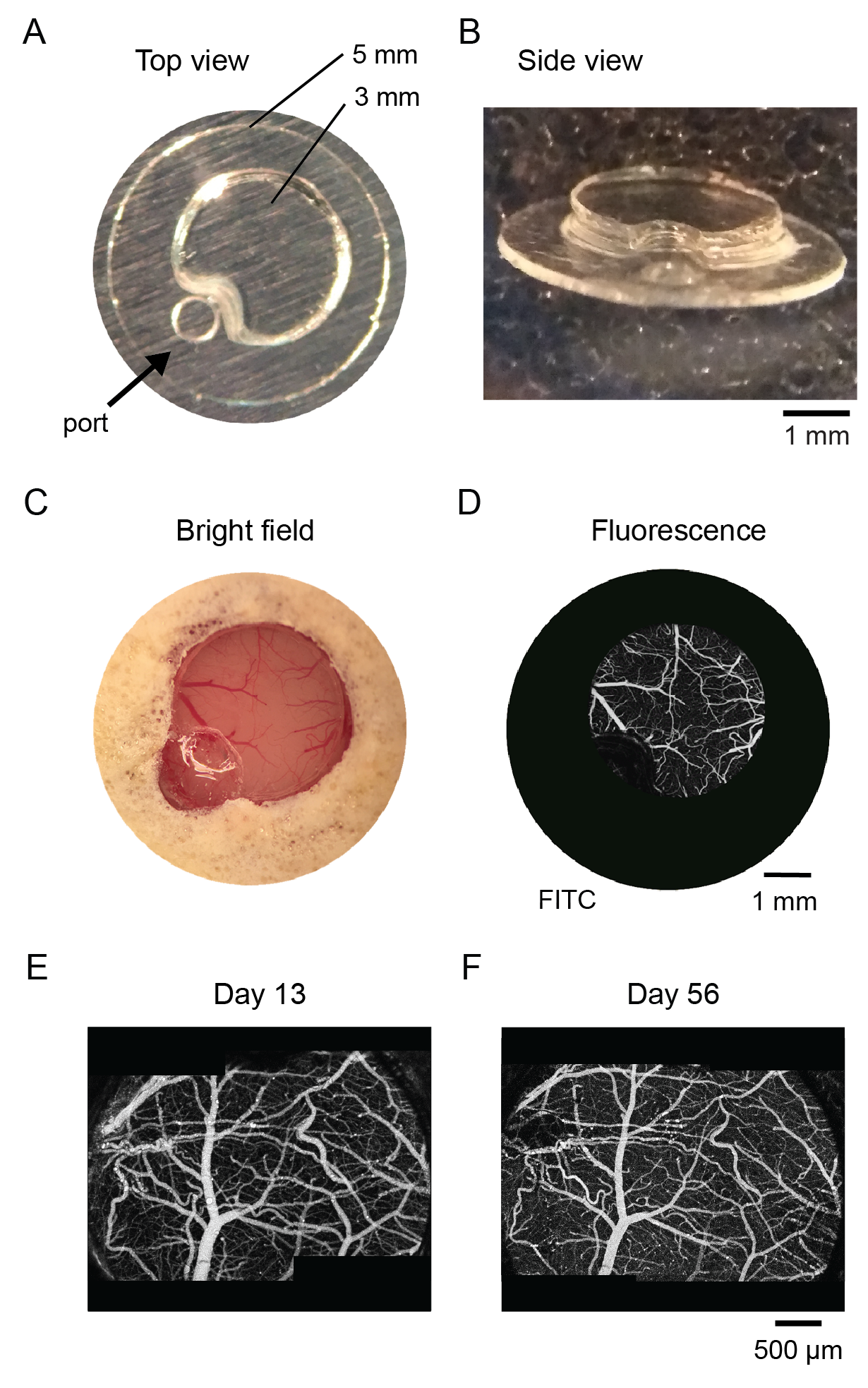

Supplement: S1 Fig — (A) Top view of the window before filling the injection port with silicone. There are three 3-mm glass coverslips stacked together and glued to a 5-mm glass coverslip. The 3-mm stack is beveled to guide the pipette at an angle through the port. (B) A side view of the window; the port is filled with silicone. (C) A top view of an implanted window; surface blood vessels are visible under the glass. (D) An image of surface vasculature within the same window calculated as a maximum intensity projection (MIP) of a 2-photon image stack 0–300 μm in depth using a 5× objective. Individual images were acquired every 10 μm. Fluorescence is due to intravascular FITC. (E) Consecutive images obtained 43 d apart demonstrate the stability of surface vasculature over time. (TIF) [file pbio.3001440.s003.tif]

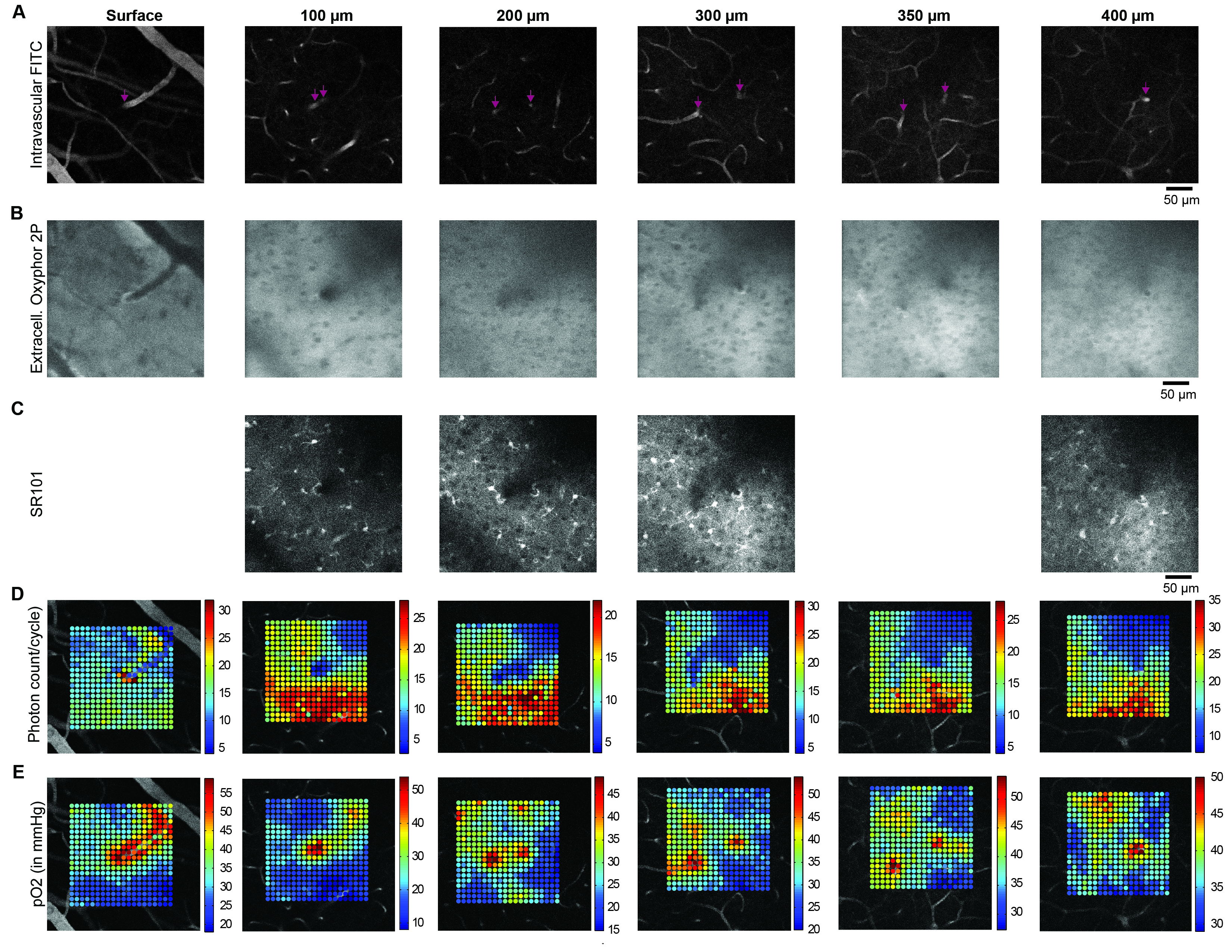

Supplement: S2 Fig — (A) Intravascular FITC images for 6 imaging planes (cortical surface, 100 μm, 200 μm, 300 μm, 350 μm, 400 μm). Arrows point to a diving arteriole that branches into 2 between 100 and 200 μm. (B) Phosphorescence images for each of these planes. (C) SR101 images; for 2 of the planes SR101 images were not acquired. (D) A square measurement grid of 20 × 20 points for each of the imaging planes. Photon counts are superimposed on corresponding vascular FITC images. (E) Calculated pO2 values superimposed on corresponding vascular FITC images. (TIF) [file pbio.3001440.s004.tif]

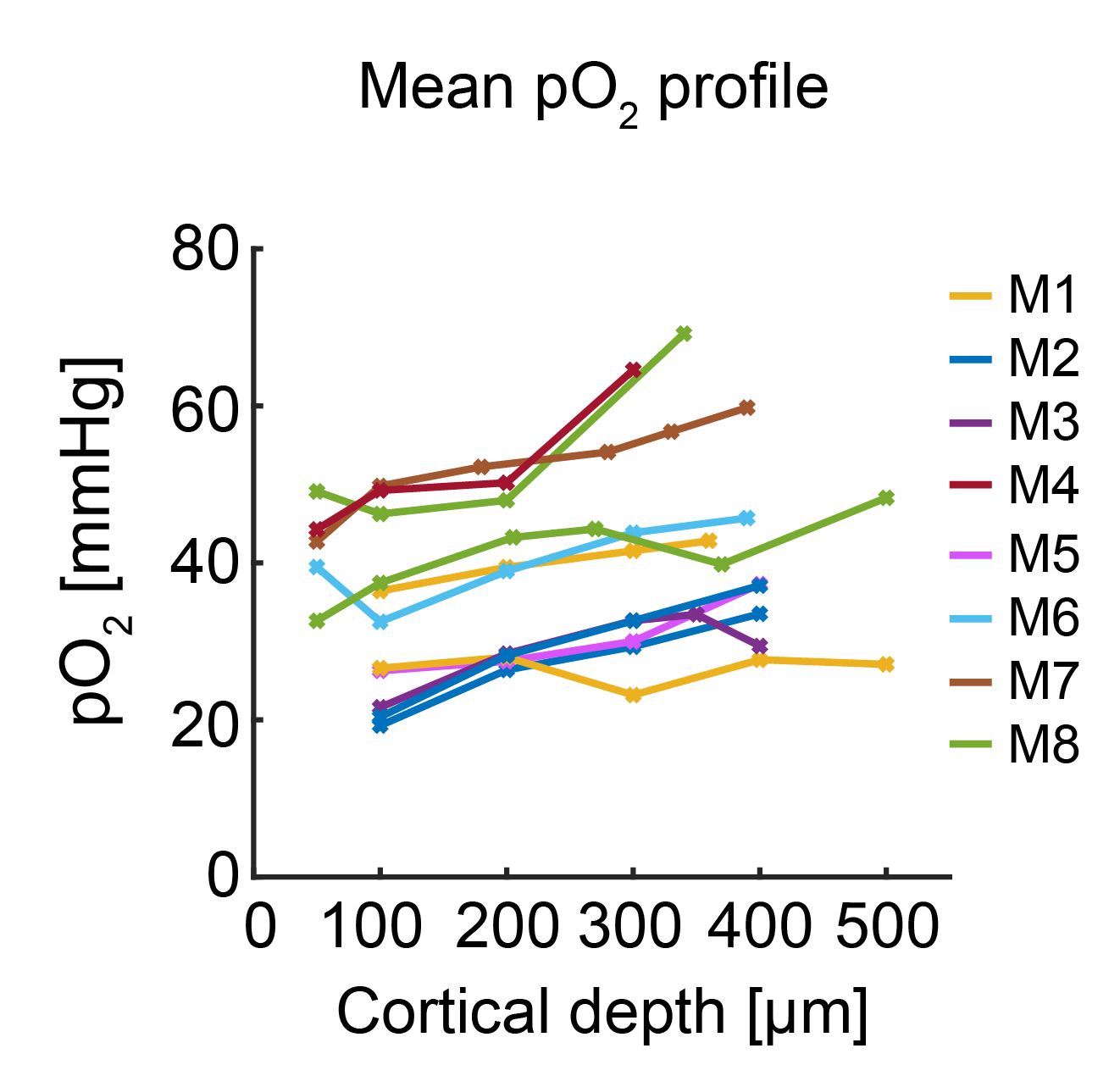

Supplement: S3 Fig — Mean pO2 calculated by averaging all points measured at the same cortical depth, plotted as a function of depth; each line corresponds to a set of measurements acquired along 1 diving arteriole. Subjects are color-coded; in 3 cases, 2 arterioles were measured per subject. Note that these values do not represent the mean tissue pO2 because they are biased towards highly oxygenated periarteriolar regions (all measurement grids in this study were centered around penetrating arterioles). Numerical values are provided in S1 Data (sheet S3). (TIF) [file pbio.3001440.s005.tif]

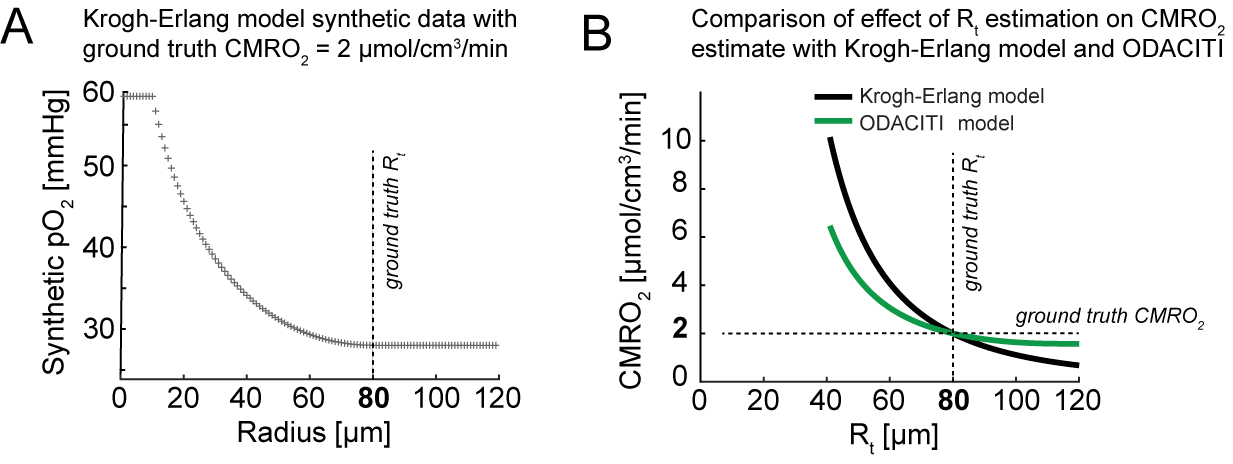

Supplement: S4 Fig — (A) Synthetic pO2 data based on the Krogh–Erlang model (CMRO2 = 2 μmol cm−3 min−1, Rt = 80 μm, Pves = 60 mmHg, Rves = 10 μm) are plotted against the distance from the arteriole. A constant pO2 is assumed for r > Rt. (B) The estimated CMRO2 after applying ODACITI (green) and the Krogh–Erlang model (black) to the synthetic data in (A) as a function of different assumed Rt values (the true Rt = 80 μm). Numerical values for (A) and (B) are provided in S1 Data (sheets S4A and S4B). (TIF) [file pbio.3001440.s006.tif]

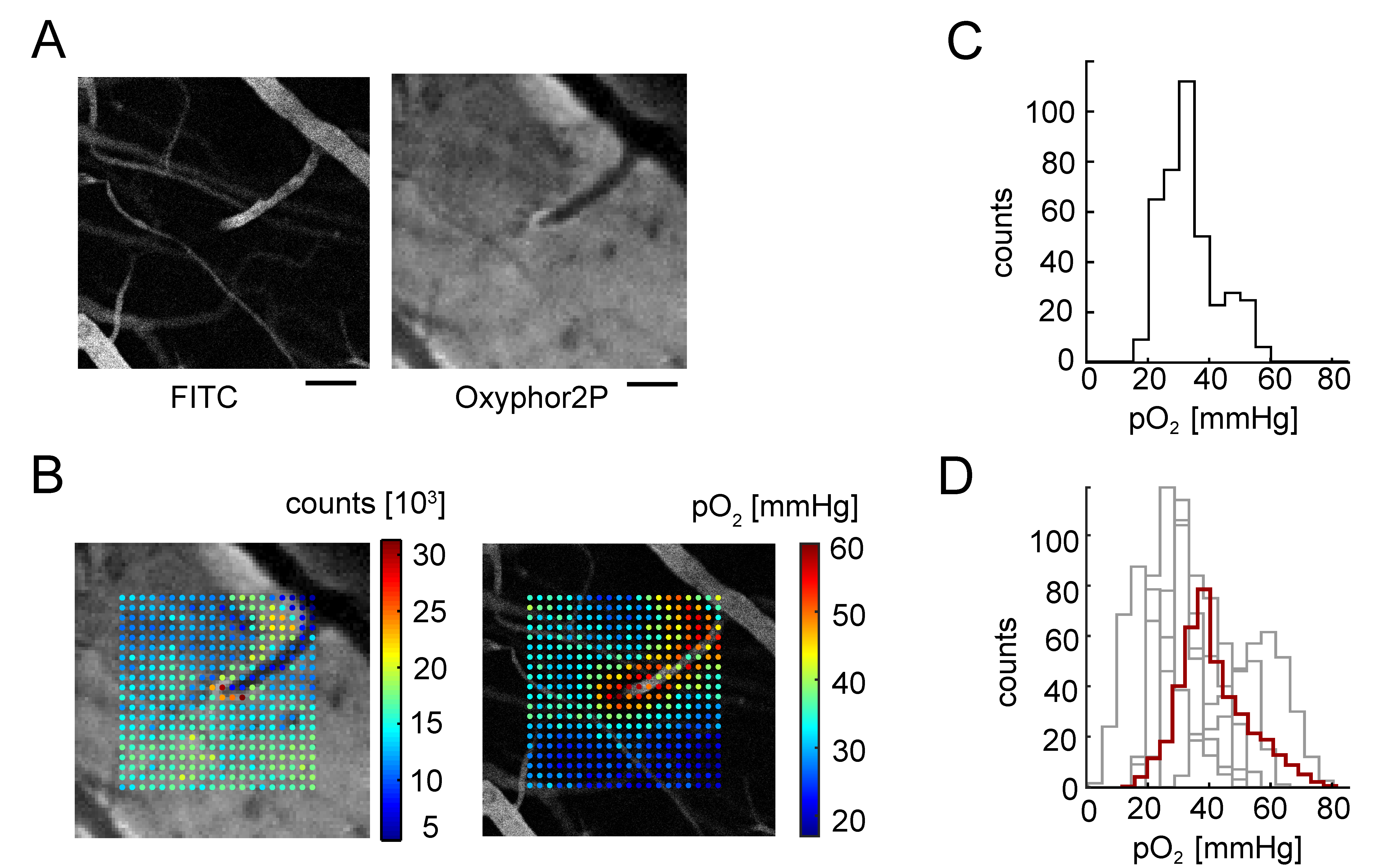

Supplement: S5 Fig — (A) Intravascular FITC image of a small surface arteriole (left) and a corresponding phosphorescence image (right). (B) A square measurement grid overlaid on the FITC image from (A). Left: Photon counts. Right: Calculated pO2 values. Scale bar = 50 μm. (C) Histogram of pO2 values corresponding to (B). (D) Overlaid histograms from each surface measurement plane (corresponding to individual arterioles, gray) and superimposed average (red). Numerical values for (C) and (D) are provided in S1 Data (sheets S5C and S5D). (TIF) [file pbio.3001440.s007.tif]

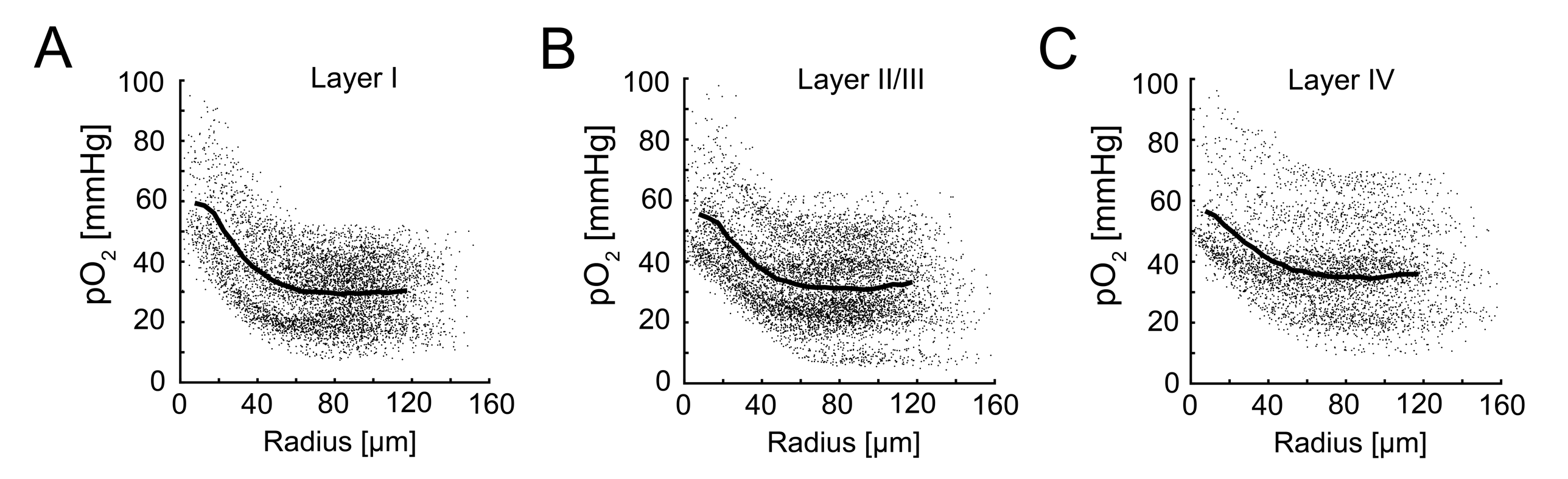

Supplement: S6 Fig — (A) All data points included in the CMRO2 estimation in Fig 3; the mean (calculated using 2.5-μm binning) is superimposed in thick black. (B) The same as (A) for layer II/III. (C) The same as (A) for layer IV. Numerical values for (A–C) are provided in S1 Data (sheets S6A–S6C). (TIF) [file pbio.3001440.s008.tif]

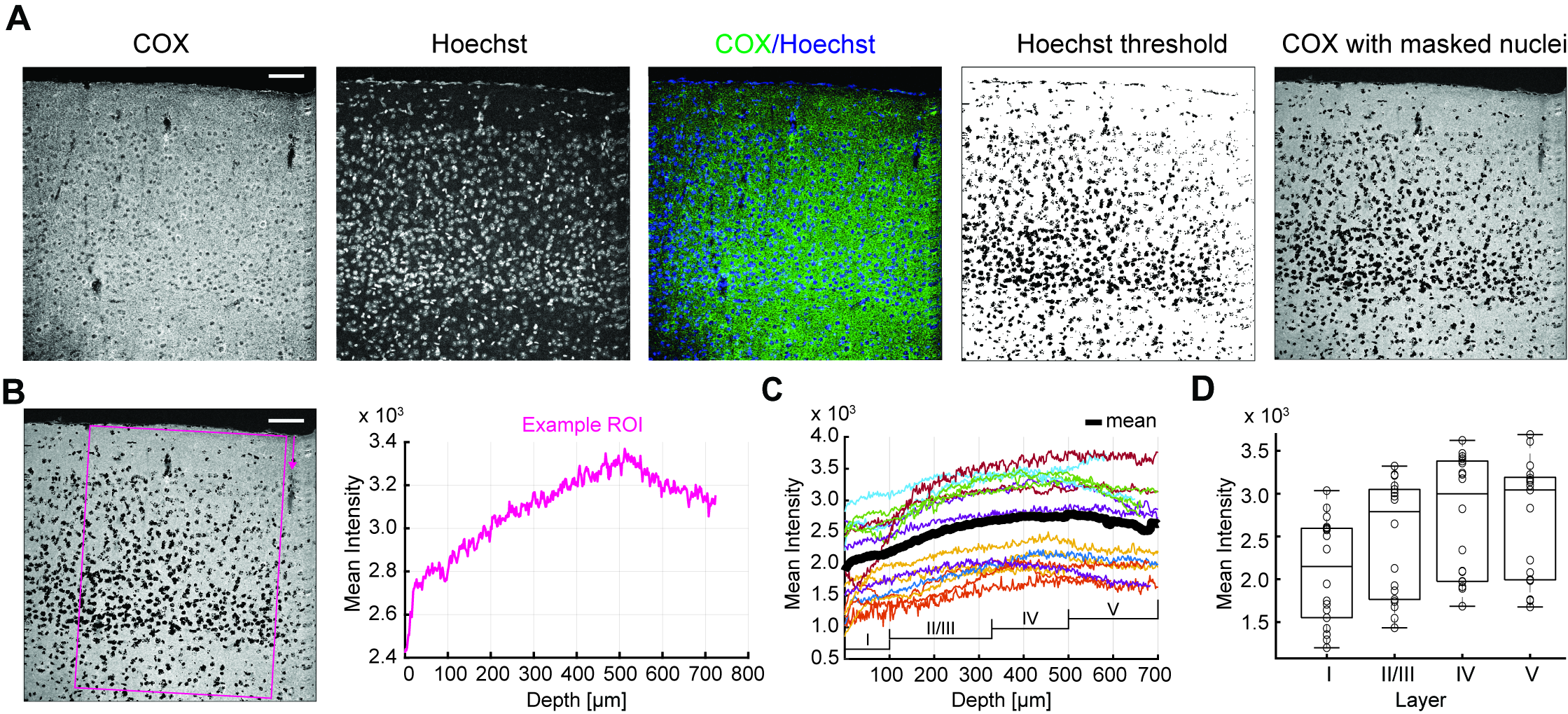

Supplement: S7 Fig — (A) From left to right: 50-μm thick cortical section immunolabeled for COX; cell nuclei (Hoechst); overlaid COX (green) and Hoechst (blue); thresholded Hoechst image; masked COX image used for quantification. Scale bar is 100 μm. (B) For COX quantification, we defined a rectangular ROI (pink) aligned with the cortical surface (left). Then, we sliced the ROI in 1-μm slices in the horizontal (laminar) direction and averaged the mean intensity within each slice (outside the Hoechst mask), resulting in the laminar intensity profile shown on the right. Scale bar is 100 μm. (C) Overlaid laminar intensity profiles for 18 ROIs (color-coded) from 4 brains. The mean is shown in thick black. (D) Boxplot of data shown in (C) sorted by cortical layer as indicated in (C). Numerical values for (C) and (D) are provided in S1 Data (sheets S7C and S7D). (TIF) [file pbio.3001440.s009.tif]

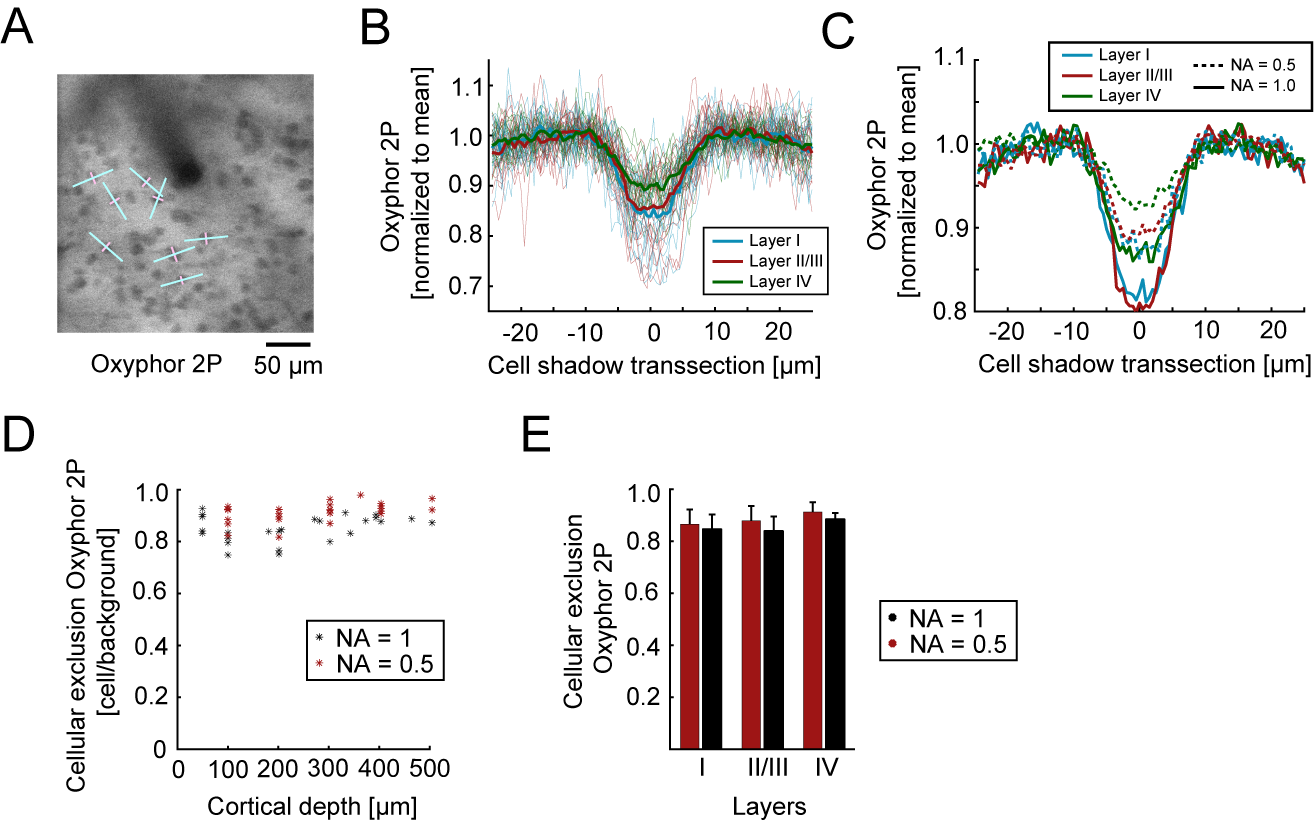

Supplement: S8 Fig — (A) For each imaging plane, the 8 most delineated (dark) cells were manually selected from phosphorescence intensity reference images. A 50-μm line was drawn across each cell (cyan) to extract an intensity profile. (B) The intensity profiles across the 8 cells were averaged and normalized to the mean intensity of neuropil (10–20 μm from center). The cortical layers are color-coded, and the mean of each cortical layer is overlaid in a thick line. (C) Comparison of data acquired with an objective with NA = 1.0 (solid lines) and NA = 0.5 (dotted lines). (D) Quantification of SBR as the mean intensity within cells (0–5 μm from center) divided by the surrounding tissue intensity (10–20 μm from center). Each point represents 1 imaging plane. The data are color-coded by the objective NA. (E) The same data as in (D) sorted by cortical layer (mean + SD). Numerical values for (B–E) are provided in S1 Data (sheets S8B–S8E). (TIF) [file pbio.3001440.s010.tif]

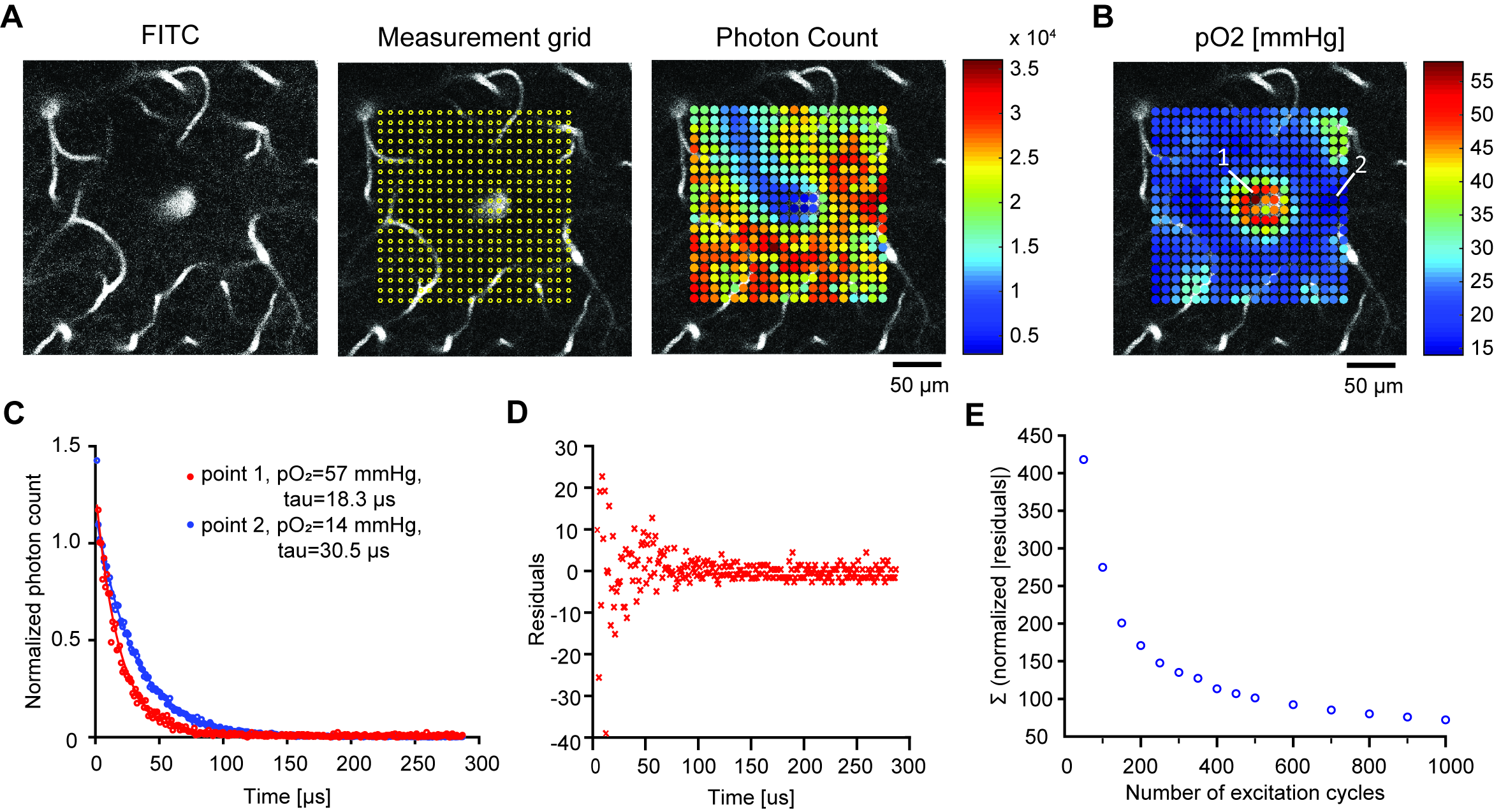

Supplement: S9 Fig — (A) Left: An imaging plane 100 μm below the surface. Fluorescence is due to intravascular FITC. Middle: A grid of measurement points overlaid on the FITC image. Right: Photon count overlaid on the FITC image. (B) pO2 values overlaid on the FITC image from (A). (C) Phosphorescence decays for 2 points labeled in (B). Data (points) and fit (lines) are overlaid. Lower pO2 corresponds to slower decay (blue). (D) Residual error over time for “point 1” from (C). (E) Residuals plotted against the number of excitation cycles. For each time bin, residuals were calculated as a sum of fractional differences: First, we computed the absolute value of the difference between the data and the single exponential fit. Next, we normalized this value by that of the fit. Finally, we summed the normalized fractional differences over the time bins. The plot shows an average across all 400 points in the measurement grid shown in (A) and (B). These results are in general agreement with quantification of the error in pO2 estimation as a function of the number of excitation cycles, which was done in our prior study [18]. Numerical values for (C–E) are provided in S1 Data (sheets S9C–S9E). (TIF) [file pbio.3001440.s011.tif]

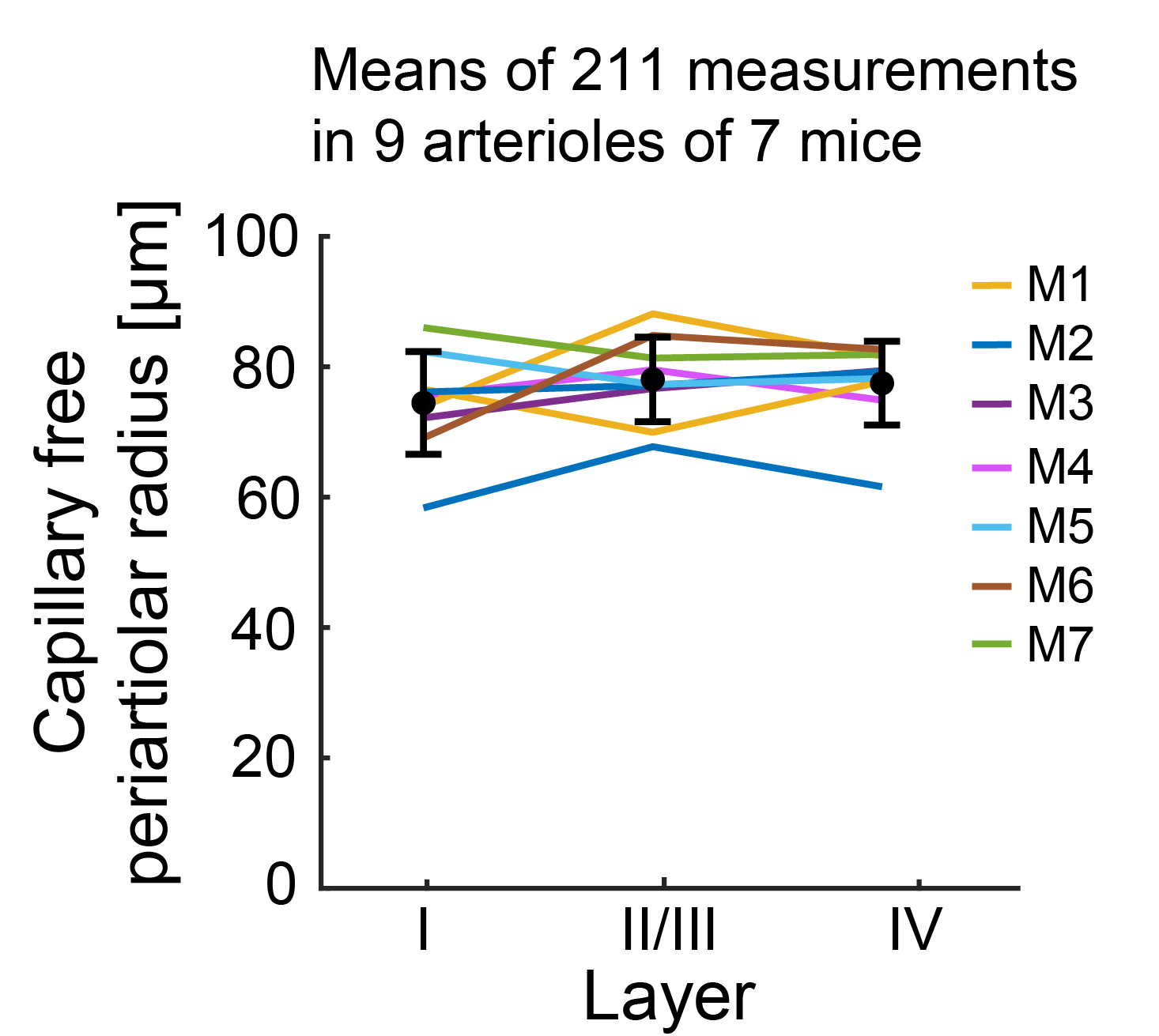

Supplement: S10 Fig — Rt was estimated from vascular FITC images as the average radial distance from the center of the arteriole to the nearest capillary within the segmented ROIs that were used for CMRO2 estimation. Data are color-coded by subject. Numerical values are provided in S1 Data (sheet S10). (TIF) [file pbio.3001440.s012.tif]
