# Supplementary material for: Baseline oxygen consumption decreases with cortical depth
Source: PLoS Biol. 2022 Oct 27;20(10):e3001440. doi: 10.1371/journal.pbio.3001440 (PMC9642908; doi:10.1371/journal.pbio.3001440)
Supplement: Attachment — Submitted filename: Rebuttal_082022.docx [file pbio.3001440.s013.docx]

Dear Editor,

Thank you very much for your consideration of our manuscript PBIOLOGY-D-21-02613 entitled "*Microscopic Quantification of Oxygen Consumption across Cortical Layers.*" We are encouraged by the overall evaluation that our study is “well executed” and “important” presenting “a practical solution to solve the [CMRO_2_ measurement] problem that will certainly move our understanding forward.” We have carefully revised the manuscript to address the Reviewers' comments, major and minor, and include a revised article where new text is in red.

Please find a point-by-point response to the Reviewers’ comments below.

We look forward to receiving your final decision.

Sincerely,

Anna Devor

**Editorial comments**

*1. The reviewers note issues with the model and simulations, high variability in the data related to a small sample size, and limitations with the contrast during deep measures which collectively undermine the strength of the conclusions. Moreover, Reviewer 1 has commented that you should compare the CMRO2 against other measures, such as mitochondrial density.. We would expect a revised manuscript to add more mice, to compare the measures of CMRO2 against other measures such as mitochondrial density, to clarify issues around the model.*

We have added 4 more mice to increase the overall sample size to 8 animals, 11 arterioles, 51 measurement planes. We also have performed post-hoc labeling for cytochrome oxidase in 4 brain and include labeling quantification in **Supplementary Figure 7**. We address comments related to model/simulation and decrease of imaging contrast with depth below in our response to Major Comment #1 of Reviewer 2 and Comment #1 of Reviewer 3, respectively.

*2. As a note, after discussion with the Academic Editor, we do not think that you need to perform measures during stimulation, as suggested by Reviewer 1 and discussed by Reviewer 3. However, without testing CMRO2 under stimulation, we agree with Reviewer 1 that the relevance of your findings to fMRI is less clear. Therefore, the motivation for the study should be changed to de-emphasize the link to fMRI and to instead highlight the implications of this work to advancing understanding of cortical physiology/developing the experimental and analytical models used here.. We would expect a revised manuscript.. to shift the motivation/intro/discussion.*

We have deemphasized the relevance to fMRI and expand the motivation for our study and discussion of our findings in the context of basic understanding of cerebral energy consumption. We have revised the relevant text as follows:

*Abstract, the last sentence:* “Our study provides the first quantification of microscopically resolved CMRO2 across cortical layers as a step towards better understanding of the brain energy metabolism.”

Introduction, we removed motivation related to fMRI in the beginning of the 1^st^ paragraph and revised the last sentence of this paragraph: “Experimentally addressing this hypothesis is important for the basic understanding of cerebrocortical energetics.”

In Discussion, we now bring up fMRI in the last paragraph strictly with respect to extending our approach to future stimulation studies:

“Knowing layer-specific CMRO_2_ in cerebral cortex is important for better understanding of the normal brain physiology as well as pathophysiology in diseases that affect cerebral microcirculation (Berthiaume et al., 2018; Iadecola, 2016, 2017; Pantoni, 2010; Zlokovic, 2011). The baseline CMRO_2_ also affects the Blood Oxygenation Level Dependent (BOLD) functional Magnetic Resonance Imaging (fMRI) signals altering the hemodynamic response for the same neuronal reality [1-5]. In the future, extending our approach to stimulation studies would help informed interpretation and modeling of high-resolution BOLD signals enabled by recent technological developments [6-9], bringing noninvasive imaging one step closer to the spatial scale of local neuronal circuits [8, 10-16].”

**Reviewers’ comments**

**Reviewer #1**

*Machler et al. made quantitative measurements of oxygen concentration around a penetrating artery in multiple layers of brain tissue. They use the results to fit a model of oxygen delivery/consumption that takes into account the role of O2 delivered by the capillaries distant from the artery, and find that the baseline CMRO2 is lower in the deep layers. This is motivated by the need to understand laminar fMRI signals, and where it is important to know CMRO2 on a layer-by-layer basis. The model is simple yet plausible. The data is nice, but very minimal. The experiments lack functional activation, as CMRO2 is known to change and the laminar-level differences might change drastically from rest. Without these key experiments, the impact of this manuscript is substantially lower.*

We thank the Reviewer for their positive evaluation of the data quality and plausibility of the model. We have added 4 more mice to increase the overall sample size to 8 animals, 11 arterioles, 51 measurement planes. Other issues are addressed in the point-to-point response below.

***Major issues***

*1. The authors motivate their study by saying that the quantitative measurements of O2 levels - combined with models of O2 consumption in the tissue - are key input into understanding laminar fMRI. I don't think the results are meaningful for laminar fMRI for several reasons. First, I am not sure the region that the authors simulate will even contribute to the BOLD signal. The hemoglobin is saturated in the arteries, and the region they are simulating is largely devoid of capillaries and totally devoid of veins, whose higher deoxyhemoglobin concentrations will generate contrast. So BOLD fMRI is effectively blind to this region.*

We have removed fMRI as the main motivation for the study. We also would like to clarify that CMRO2 estimated from the periarteriolar region is likely to be representative of that of tissue fed by the surrounding capillary bed as well as in a perivenular region *as long as we stay within the same cortical column and layer*. We detail scientific premise behind this argument in response to Reviewer’s Comment #2 below.

*Second, when using laminar fMRI in the context of a task, both the resting and the task-evoked signal will depend on CMRO2, and CMRO2 will change with the task, potentially in layer-by layer fashion. Measuring CMR02 at rest is only half of the metabolism story. While we know a fair bit about differences in CBF and vascular density across layers, one needs to know how CMRO2 changes with stimulation in order to say anything about the basis of laminar BOLD signals. Without some comparisons to a sensory-evoked condition, I don't see these measurements as being useful to the laminar fMRI community.*

We agree with the Reviewer that “measuring CMRO2 at rest is only half of the metabolism story.” While a stimulation/task study is currently underway in our laboratory, the results at this point are too preliminary to be included in the current manuscript. Our preference would be to publish these results in due time as a follow up paper. For the current manuscript, we have followed the Editorial guidelines to deemphasize the relevance to fMRI and expand the motivate our study in the context of the basic understanding of cerebral energy consumption. Please, see our response to Editorial Comment #2.

*2. The model and the data fitting assume that CMRO2 is constant at all distances from the arteriole.  I don't think this likely, and the authors should consider the alternative hypothesis.  One might expect that more metabolically demanding cell types (interneurons) to be clustered around the arteriole to take advantage of the higher O2 concentrations there.  The authors could have relaxed this constraint and estimated how the observed O2 gradients might be impacted by CMRO2 variation with distance.*

The Reviewer correctly points out that we treat CMRO2, estimated from periarteriolar pO2 gradients, as representative for a region extending beyond the measurement grid. For example, we assume that CMRO2 is space-invariant between a supplying arteriole and a draining venule as long as we stay within the same cortical column and layer. This assumption is based on the following lines of evidence:

a. The location of diving arterioles does not map onto the structure of cortical columns and septa (regions in between columns with low-density cytochrome staining) in the whisker-barrel cortex. This lack of spatial correspondence was first described by Tom Woosley [17] and recently confirmed by David Kleinfeld’s lab using contemporary microscopy and reconstruction tools [18]. Blinder et al. concluded that “the arrangement of the penetrating vessels that source and drain blood to the microvasculature, as well as the resulting calculated patterns of flow, bear no correspondence with cortical columns.”
b. The mitochondrial/cytochrome density *within a column* does not co-vary with distance from diving arterioles. E.g., a study in the visual cortex reported that microvascular lobules and cytochrome oxidase patches within a cortical layer “bore no mutual relationship” [19].
c. Quantification of mitochondrial ATP production as a function of pO2 by David Wilson [20] suggests that production of ATP should not be affected within the range of tissue pO2 values reported here as well as in previous studies in healthy adult mice [21, 22].
d. Another study from the Kleinfeld lab concluded that “neuronal and vascular densities do not track each other on the 100 μm scale of cortical lamina” [23]. Although this study did not specifically examine interneurons, there is no evidence that (any types of) interneurons are clustered around arteries. Importantly, *O2 consumption occurs locally at mitochondria that are present not only in cell somas but also along the dendrites* that often extend throughout the width of a cortical column and beyond. Therefore, even when a cell body of an interneuron happens to be located next to an arteriole, the dendrites will span a large volume encompassing the capillary bed and venules. This is evident from many studies where individual neurons of various types have been labeled and morphologically reconstructed, e.g., works from Julius Zhu’s lab [24].
e. The above-mentioned evidence is also consistent with a central idea in developmental neuroscience that formation of neuronal circuits is governed by their computational efficiency and should not be constrained by supporting systems. In the words of Tom Woolsey, during development “blood vessels grow and adapt to supply the local metabolic requirements of neural modules in the brain” [25] such that the physical location of neurons in healthy brain is independent from the location of feeding arterioles.
f. Related to the previous point, our recent intravascular study shows that baseline O2 extraction fraction in SI of awake mice is 30-40% leaving a safe margin for additional extraction from capillaries and venules during neuronal activation even downstream the capillary path and in the perivenular region [26].

Collectively, this body of evidence provide a strong scientific premise for our assumption of space-invariant CMRO2 **at the local scale of cortical columns and lamina**. We have revised the Discussion section (p. 15-16) to flesh out this argument:

“The CMRO_2_ estimate derived from a periarteriolar region is likely to represent CMRO_2_ in the surrounding tissue fed by capillaries as well as in neighboring perivenular regions, as long as we stay within the same cortical column and layer. Several convergent lines of evidence support this thesis. First, it has been shown that the location of penetrating arterioles does not map onto the structure of cortical columns and septa in the barrel cortex [17, 18]. Second, the mitochondrial/cytochrome density within a column does not co-vary with distance from penetrating arterioles [19]. Third, quantification of mitochondrial ATP production as a function of pO_2_ [20] suggests that the production should not be affected within the range of tissue pO_2_ values reported here as well as in previous studies in healthy adult mice [21, 22]. Fourth, a microscopic reconstruction study has concluded that neuronal somas and vascular densities do not track each other on the 100-μm scale of cortical lamina [23]. Further, O_2_ consumption occurs locally at mitochondria that are present not only in somas but also along the dendrites that often extend throughout the width of a cortical column and beyond [24, 27]. Therefore, even when a soma happens to be located next to an arteriole, the dendrites can span a large volume encompassing the capillary bed and perivenular regions. Finally, formation of neuronal circuits during development is not constrained by location of feeding arterioles (Wang et al 1992), and baseline O_2_ extraction fraction in SI of awake adult mice is only 30-40% leaving a safe margin for additional extraction from capillaries and venules during neuronal activation even in-between downstream capillaries and in the perivenular region [26]. Collectively, this body of evidence provide a strong scientific premise for our assumption of space-invariant CMRO_2_ at the local scale of cortical columns and lamina.”

*3. There are no quantifications or direct comparisons to previously published studies of laminar mitochondrial density, which could be a key indicator of actual CMRO2, and which would be sensible thing to test the model against. To start, one might expect that CMRO2 to linearly follow mitochondrial density. The Weber (2008 paper) is for primate visual cortex and the Land/Simons (1985) paper is not quantitative.*

In the original version of our manuscript, we cited “A Quantitative Study on the Distribution of Mitochondria in the Neuropil of the Juvenile Rat Somatosensory Cortex” [28] that reports laminar mitochondrial density based on segmented mitochondria in electron microscopy sections. This study is consistent with findings by many others (including Bruno Weber) that mitochondrial density increases from layer I to layer IV. In response to this comment, we have performed fluorescent labeling for cytochrome oxidase in 4 mice matched by strain and age to the ones used for *in vivo* imaging. These results and quantification are now included in **Supplementary Figure 7** that we append below. We also added a corresponding section to Methods titled “Immunocytochemistry.” In agreement with the literature, our results show an increase in cytochrome oxidase density from layer I to layer IV.

**Supplementary Figure 7. Quantification of laminar cytochrome oxidase (COX) density**

**A.** From left to right: 50-μm thick cortical section immunolabeled for COX; cell nuclei (Hoechst); overlaid COX (green) and Hoechst (blue); thresholded Hoechst image; masked COX image used for quantification. Scale bar is 100 μm.

**B.** For COX quantification, we defined a rectangular ROI (pink) aligned with the cortical surface (left). Then, we sliced the ROI in 1-μm slices in the horizontal (laminar) direction and averaged the mean intensity within each slice (outside the Hoechst mask) resulting in the laminar intensity profile shown on the right. Scale bar is 100 μm.

**C.** Overlaid laminar intensity profiles for 18 ROIs (color-coded) from 4 brains. The mean is shown in thick black.

**D.** Boxplot of data shown in (C) sorted by cortical layer as indicated in (C).

***Minor***

*1. Data will be shared upon publication- this does not conform to pls biology guidelines, needs more detail about the sharing procedure. The code for the model should be shared as well.*

We concur with the Journal’s sharing policy. We have deposited the dataset corresponding to this study to [Brain Imaging Library](https://www.brainimagelibrary.org/) that is a public repository funded by the BRAIN Initiative. The dataset ID is pending BIL format verification. This dataset will become publicly available when the paper is accepted for publication. The code will be made available through [GitHub](https://github.com/).

*2. P 20 "silicon port" I think this should be silicone?*

We fixed this spelling mistake, thank you.

**Reviewer #2**

*In their manuscript titled: "Microscopic Quantification of Oxygen Consumption across Cortical Layers", Mächler, Fomin-Thunemann present measurements of CMRO2 from the Layer I to the layer IV of the somatosensory cortex S1 of awake but unstimulated mice. They combined Two-photon Phosphorescence lifetime imaging with a recent, red-shifted oxygen sensor (Oxyphor 2P) and an innovative modeling approach to extract baseline consumption levels from oxygen consumption gradients present around pial arterioles. They report increased baseline oxygen levels in the layer IV (as compared to more superficial layers) as well as a reduced baseline oxygen consumption level at rest. This result is in contrast with established and published higher energy demands in the layer IV from Cytochrome oxidase staining and capillary densities studies. They conclude that transient and rapid increase in oxygen consumption upon sensory stimulation must account for this discrepancy.*

*The quantification of baseline cerebral oxygen demand is an important endeavor which will yield many benefits for understand brain energy budgets and its regulation by biological processes. I applaud the efforts of the author in tackling this important question by integrating complex and modern instrumentation with modeling. Neuro-vascular units are tightly integrated components and precise compartmentalized and appropriately parametrized models are needed to push the field forward. Cerebral CMRO2 is a key variable in those models but has been hard to measure in vivo. This study presents a practical solution to solve this problem and will certainly move our understanding forward.*

*In addition, the manuscript is clearly written and easy to read. It also references the relevant literature. All figures and captions are properly captioned. I commend the author for this clear presentation.*

*While I have no concerns about the importance of this work, my main comments are methodological to make sure that the measure of CMRO2 was properly validated so that future work can rely upon it.*

We thank the Reviewer for appreciating the significance of our study. We are also happy to hear that our modeling approach is “innovative”, and presentation is clear.

***MAJOR COMMENTS***

*1. Impact of baseline oxygen supply from nearby capillaries.*

*The authors introduced a modification of the standard Krogh-Erlang model of O2 diffusion to account for the contribution of the capillary bed. As I understood it, in the ODACITI model, a key assumption is that for r<Rt, all oxygen molecules are provided by the pial arteriole and for r>Rt, all oxygen are provided by the capillary bed. As a result, oxygen flux at the boundary r=Rt is null. This hypothesis confused me somehow. In real tissue, shouldn't there be a transitional region where both capillary beds and the arteriole contributes? It seems to me like the capillary beds would need to have quite a complex geometry to satisfy this strict boundary condition. Presumably contribution from arteriole to CMRO2 gradually decrease with distance and capillary bed gradually takes over.*

*This assumption is important because for the measured CMRO2 to be accurate, the measured gradient from the pial arteriole needs to account for 100% of the baseline consumption. If for example, nearby capillary beds would contribute to this baseline consumption, that will cause an underestimate of CMRO2, thereby impacting the reported decrease in oxygen consumption in the layer 4.*

We apologize for not presenting the model clearly. While, according to ODACITI model, the capillary bed indeed starts at r=Rt, the model is not imposing a restriction that tissue inside the r<Rt radius is strictly supplied by oxygen from the arteriole. As the Reviewer implied, ODACITI indeed allows for some O2 supplying tissue within the Rt radius to be drawn from the capillary bed. While most of the r<Rt region is strictly supplied by the arteriole, there is indeed a transitional region inside r<Rt (at some r=R0<Rt, which is close to Rt) which is strictly supplied by the capillary bed. The meaning of ‘transitional region’ here is that it is supplied by the capillary bed, but it contains no capillaries. Consequently, there is a cylindrical boundary at r=R0 inside Rt through which a net oxygen flux is zero. Note that when R0=Rt, ODACITI scales back exactly to the Krogh-Erlang model for r in between Rves and Rt. Since the goal is to recover CMRO2, the equations are specifically tailored to enable robust fitting of CMRO2 and not to explicitly fit for the radius of the R0 boundary, and R0 is a hidden parameter. Please note that inside the tissue cylinder r<R0, the PO2 gradient from the diving arteriole indeed accounts for 100% of the baseline O2 consumption, as the Reviewer mentioned in the question. Therefore, we believe that our modeling of capillary bed contribution to tissue consumption inside the cylinder Rt should not lead to systematic underestimation of CMRO2.

We have revised Results, “ODACITI model for CMRO2 extraction”, to improve clarity and emphasize the fact that ODACITI allows for some of the O2 consumed within the Rt cylinder to be drawn from the capillary bed (p. 8):

“In the Krogh-Erlang model, a blood vessel – approximated as an infinitely long cylinder with the radius $r=R_{ves}$ – has a feeding tissue territory with a radius $r=R_{t}$. Inside this cylinder, all O_2_ is derived from the vessel at the center. Therefore, according to the Krogh-Erlang model, tissue pO_2_ will monotonically decrease with *r* while moving away from the vessel until we reach $R_{t}$ where the total O_2_ flux through the cylindrical surface with radius *R_t_* is zero ($dpO_{2}/dr=0)$. Outside of this cylinder, the predicted pO_2_ for $r>R_{t}$ has no physiological meaning as the model is defined for $r\leq R_{t}$. Previously, we treated *R_t_* as the radius of the periarteriolar space devoid of capillaries [29].

The new model, which we call ODACITI (**O**_2_ **D**iffusion from **A**rterioles and **C**apillaries **I**nto **TI**ssue), does not impose a restriction that tissue inside the *r<R_t_* radius is strictly supplied by O_2_ from the center arteriole. Rather, it allows a “transitional region” inside the *Rt* cylinder (at *R_0_<r<R_t_*) which is strictly supplied by the capillary bed. Because *R_t_* is the radius of capillary-free periarteriolar space, the transitional region is supplied by the capillary bed but contains no capillaries. Consequently, there is a cylindrical boundary at *r=R_0_* inside *R_t_* through which a net O_2_ flux is zero (**Fig. 2A**). For any cylindrical surface with radius *r* between $R_{ves}$ and $R_{t}$, the total O_2_ flux in ODACITI represents a difference between O_2_ diffused from the vessel out and O_2_ consumed in the inner tissue cylinder (see **Methods**). ODACITI solution is also defined for $r>R_{t}$, where CMRO_2_ is exactly equal to O_2_ delivery rate of the capillary bed. In this way, ODACITI allows including data points beyond $R_{t}$ improving robustness of the fitting procedure and can better account for the influence of the capillary bed as compared to the Krogh-Erlang model (see **Methods**).”

We also revised one paragraph in Methods, p. 24, to emphasize the same issue:

“Here, we derive a specific solution to the forward problem of this partial differential equation assuming a cylindrical central arteriole with the radius *r=R_ves_* and a region around the arteriole void of capillaries within a radius *r=R_t_* similar to the Krogh-Erlang model of O_2_ diffusion from a cylinder [30]. In contrast to the Krogh-Erlang model, we assume that, while most of the *R_ves_<r<R_t_* region is supplied by the arteriole, there exists a transitional region inside the *R_t_* cylinder (at *R_0_<r<R_t_*) which contains no capillaries but is supplied by the capillary bed. With these assumptions, we can derive an analytic solution to **Eq. 4** that we describe below.”

*The authors performed some simulation to try to test this caveat but I felt the simulation was a little limited. When does this assumption break? The author mentioned some parameters of the simulation matched real dataset (Page 10. "which is a typical size of the region around diving arterioles void of capillaries in mouse cerebral cortex.") but I was not sure whether this was a qualitative match or if some measurement of typical capillary density was used.*

Our apologies for the lack of clarity creating this confusion. As we explain above, ODACITI does allows a transitional region. The FEniCS simulations with additional pO2 sources that Reviewer is referring to were performed as means for validation of our segmentation algorithm. Regarding parameters derived from data, we used reference 2-photon vascular images to measure the radial distance from the center arteriole to the nearest capillary within segmented regions of interest. We now include these measurements within **Supplementary Figure 10** that we append below.

**Supplementary Figure 10. Experimental measurement of R_t_**

$R_{t}$ was estimated from vascular FITC images as the average radial distance from the center of the arteriole to the nearest capillary within segmented ROIs which were used for CMRO_2_ estimation. Data are color-coded by subject.

*This hypothesis also confused my interpretation and intuition of the equations.*

*Page 24: "Third, we assume that for r>Rt the rate of O2 delivery by the capillary bed is uniformly distributed and equal to the rate of tissue O2 consumption."*

*Page 25: equation 8, why does pO2 depends on the radius for r>Rt if there is a perfect equilibrium between supply and demand for r>Rt.*

*If the authors could provide more simulated controls and clarify the impact of these hypothesis on the interpretation of the data and associated equation, that will strengthen the use of this methodology in the future.*

Since we have a transitional region inside Rt that is supplied by the capillary bed, there is a small O2 flux from the capillary bed into the transitional region. However, since inside the capillary bed we have a perfect match of the tissue O2 demand solely met by the capillary bed, the extra O2 flux that supplies the transitional region appears in equations as being continuously drawn from infinity into the transitional region, which is why ODACITI equations have a dependence on r beyond Rt. This small PO2 gradient in the r>Rt region may be difficult to observe experimentally. However, this mostly flat PO2 region is outside the tissue cylinder where CMRO2 is supplied solely by the diving arteriole and which is the key for the CMRO2 estimation.

Once again, we apologize for the confusion. ODACITI does *not* rely on the assumption that for r<Rt, oxygen molecules are provided exclusively by the center arteriole.

***MINOR COMMENTS***

*2. Page 6: "within ~40 min after the injection". What was the rationale for waiting such a long time before doing measurement?* *Does the injection impacts local oxygen levels?*

The main reason for waiting 40 min was that it allowed the O2 probe to diffuse around the injection site so that, e.g., we could do measurements across layers (as we mention in Methods).

One additional consideration is that a pinprick of the cortical surface has been used in other studies as a means to induce Cortical Spreading Depression (CSD). Although we think that this outcome was unlikely (our quartz pipettes are very thin), waiting for 40 min ensured that cerebral blood flow was back to its baseline in an unlikely event of CSD. We now mention this potential confound in “Delivery of optical probes” on p. 22:

“Insertion of the pipette into the brain tissue could in principle induce Cortical Spreading Depression (CSD). Although we think that this is an unlikely possibility because the pipettes were very thin, we have not measured a DC potential during the injection and cannot rule out that CSD occurred in some cases. Previous studies have shown that in mice CSD results in a transient CBF decrease that recovers within ~30 min [31, 32]. Therefore, waiting for 40 min after the injection also ensured that CBF was back to its baseline even in an unlikely event of CSD.”

Regarding the last question about the probe depleting local O2, we have repeatedly tried to oversaturate the excitation volume, in both intravascular and tissue PO2 measurements, but never observed any signs of O2 depletion in either fitted pO2 values nor lifetime profiles.

*3. In the discussion, the importance of this absolute CMRO2 measurement for the fMRI BOLD signal is discussed. While this is a common statement in the neurovascular literature, I see more values in understanding fundamental neurovascular processes. BOLD signals are likely a mixture of tissue and blood oxygen levels and it is unclear to me how much it relates to absolute vs relative levels in oxygen consumption. Perhaps the authors could discuss in what ways they think this work will help understand BOLD more precisely.*

We hear the Reviewer. Following this comment as well as similar comments by other Reviewers and the Editor, we deemphasized the motivation of our present results by the need to understand the BOLD signal and discuss the relevance to fMRI only in the context of extending our approach to future stimulation studies. Please, see our response to Editorial Comment #2.

*4. Figure 1 is missing a few scale bars for a subset of panels. The depth used for each planar recordings on the figure is also missing.*

We inserted the missing scale bars and labels. Thank you.

*5. Figure 3. I was confused with panel B. How can the ODACITI fit predicts a small increase in pO2 if the surrounding tissue is in equilibrium with energy consumption?*

As we mention in response to Major Comment #1, the extra O2 flux that supplies the transitional region appears in ODACITI equations as being continuously drawn from infinity into the transitional region, which is seen as a small PO2 gradient in the r>Rt region. This gradient is small and not always observed experimentally, but in this example, it is present in the data and also captured by the ODACITI fit.

*6. Figure 3. Are panel E and F duplicates? If that is the case, I felt duplicating that plot was confusing.*

We apologize for the lack of clarity. Panel E shows experimental data on pO2 (mean plus/minus standard error) across cortical layers. Panel F shows ODACITI fit to the data for each layer. We have clarified the difference in the legend. In principle, we could have combined both in the same panel, but we felt that it would overcrowd the panel. Revised **Figure 3** (including data from all 8 animals) and the figure legend are appended below.

**Figure 3. CMRO2 estimation across cortical layers**

**A.** An imaging plane 100 μm below the surface; a grid of pO2 points is overlaid on the vascular image. pO2 values were interpolated along the radial yellow lines. The yellow shaded area extends along each direction until the first derivative becomes zero. Segmented ROI used for extraction of the radial pO2 profile is shown on the right. This ROI includes in addition values within the low tail of the pO2 distribution.

**B.** The radial pO2 profile extracted from (A). ODACITI fit is overlaid on the data. The fitted CMRO2 value is 1.5 μmol cm^-3^ min^-1^.

**C.** Estimated CMRO_2_ for the entire dataset of 51 planes along 11 diving arterioles in 8 subjects. Measurements along the same arteriole are connected in a line. Subjects are color-coded.

**D.** Quantification of CMRO_2_ across layers using the data in (C). Error bars show standard error calculated using a linear mixed effect model implemented in R.

**E.** Experimentally measured tissue pO2 (mean ± SEM, binned at 5 µm) across cortical layers (color-coded) as a function of distance from the arteriole. Each profile represents a grand average across subjects.

**F.** ODACITI fit for the data shown in (E) (binned at 2 µm).

*7. Supplementary Figure 6. I applaud the quantification of residual measurement error. I was not entirely sure what were the units of those plots. Ideally, the error in decay time was converted to an averaged residual error in pO2 (using the calibration curves) and panel E is in mmHg. I was not sure given the figure and caption here.*

We have done quantification of the error in pO2 estimation as a function of the number of excitation cycles in our recent study that used the same dye and experimental procedures [33]. Below we reproduced a supplemental figure from this study that shows the result. These measurements were done in a diving arteriole and surfacing venule in an awake mouse at cortical depth of 600 μm. Error bars are the standard deviation across repeated (n=3) measurements. Although this figure shows intravascular measurements, the error in pO2 as a function of the number of excitation cycles is independent of whether the dye is present in the vasculature or tissue as long as the imaging conditions (O2 probe, dark/light photon counts) are similar.

We have now included citation to this earlier work in the corresponding figure legend.

***Reviewer #3***

*In the study by Machler et al., 2PLM measurements of PO2 are made across depth of S1 in awake mice in which the recent probe Oxyphor2P was injected directly into the cortex. They find that tissue PO2 immediately in the vicinity of the arteriole does not change significantly as a function of depth (consistent with previous vascular measurements), whereas "the low tail", shows an increasing PO2 concentration with increasing depth up to L4. Using a modified version of krogh model they develop termed "ODACITI", they estimate a decrease in CMRO2 from L1 to L4 at baseline. This is surprising as it contrasts with cytochrome oxidase staining which is highest in L4.*

*Generally, the study is well executed and important for understanding O2 distribution in the cortex and for BOLD-fRMI interpretation. There are limitations to the study, many of which are addressed in the discussion.*

We thank the Reviewer for evaluation of our study as “well executed and important.”

*Comments:*

*1. A major limitation of the approach (as acknowledged by the authors) is that for the deeper measurements they begin to lose "contrast" due to excitation near the surface. This could potentially result in an artifact in which the signals appear to be smoothed out. How can we trust the measurements with this problem in mind?*

As the reviewer correctly points out, the signal-to-background ratio (SBR) in our measurements decreases with depth; this is a general limitation of 2-photon microscopy. The reason for this is out-of-focus excitation (OFE) that we acknowledge on p.16 as one of the caveats potentially leading to smoothing of the periarteriolar gradients. In response to this comment, we have included quantification of SBR of phosphorescence intensity as a function of cortical depth in a new **Supplementary Figure 8** appended below. Since the original submission, we have acquired data from 4 additional animals. In these recent experiments were used an objective with NA=1 in contrast to the original 4 animals where we used an objective with NA=0.5 (see also our response to the next question below). As can be observed from the figure, NA=1 improves SBR. Nevertheless, we have observed the same behavior of CMRO2 across layers as in the first half of this dataset. Therefore, we believe that the smoothing effect was not significant. OFE and deterioration of SBR with depth was the reason why we limited the current study to layers I-IV, although the probe was present also in layer V.

We have revised the corresponding paragraph of Discussion (p. 16) as follows:

“Our measurements were limited to cortical layers I-IV. In 2-photon microscopy, depth penetration is fundamentally limited by out-of-focus excitation [34]. Because light is scattered and absorbed by tissue, the laser power is ought to increase with depth to overcome these effects and deliver a sufficient number of photons to the focal volume. In our experiments, an increase in the number of scattered photons would increase the probability of out-of-focus excitation of Oxyphor 2P generating background phosphorescence. The lifetime of this background phosphorescence would report pO_2_ at locations outside the focal volume contributing to the experimental error and smoothing out peri-arteriolar pO_2_ gradients. In this study, we acquired data using two different objectives with NA=0.5 and NA=1. While higher NA reduced out-of-focus excitation (quantified as signal-to-background ratio (SBR) in **Suppl. Fig. 8**), we have observed the same behavior of CMRO_2_ irrespective of the NA. Therefore, we believe that the smoothing effect was not significant. In the future, a combination of 2PLM with light sculpting techniques [35] can be used to experimentally measure the amount of generated out-of-focus signal. 2PLM can also be implemented using two laser beams of different color that can be displaced in space minimizing out-of-focus excitation [36, 37].”

**Supplementary Figure 8. Estimation of signal-to-background ratio (SBR)**

**A.** For each imaging plane, the 8 most delineated (dark) cells were manually selected from phosphorescence intensity reference images. A 50-µm line was drawn across each cell (cyan) to extract an intensity profile.

**B.** The intensity profiles across the 8 cells were averaged and normalized to the mean intensity of neuropil (10-20 µm from center). The cortical layers are color-coded and the mean of each cortical layer is overlaid in thick lines.

**C.** Comparison of data acquired with an objective with NA=1.0 (solid lines) and NA=0.5 (dotted lines).

**D.**  Quantification of SBR as the mean intensity within cells (0-5 µm from center) divided by the surrounding tissue intensity (10-20 µm from center). Each point represents one imaging plane. The data are color-coded by the objective NA.

**E.** The same data as in (D) sorted by cortical layer (mean + SD).

*It is unclear why they are using a 0.5NA objective (methods, p18), instead of a higher NA objective? This would greatly increase the out of focus excitation they are trying to eliminate for the spatial precision of their measurements.*

For the original dataset, we used a headpost design that did not allow using wide objectives with high NA. Since then, we have redesigned the headpost and were able to use an objective with NA=1.0. The revised manuscript includes 4 original animals with 0.5NA and 4 additional ones with 1.0NA. The reviewer is correct predicting that higher NA would reduce OFE. At the start, it was not clear to us that this would be the case, because higher NA results in a wider cone of illumination, such that rays at the edge travel a longer path and this experience more scattering contributing to OFE. On the other hand, smaller NA reduces the collection efficiency leading to the need to increase laser power that leads to OFE. Our experimental data show that the latter effect is more significant, such that higher NA results in reduction of OFE.

*It was not entirely clear to me if the 3D distribution of capillaries was taken into consideration in the model. As L4 has higher capillary density, could this explain the higher PO2 in the low tail of the distribution in L4?*

ODACITI assumes cylindrical symmetry (similar to the Krogh-Erlang model) and does not take into account 3D capillary structure. In terms of experimental data, higher tissue pO2 in L4 is consistent with our recent intravascular study where we reported higher intracapillary pO2 in L4 compared to upper layers [26]. The reviewer is correct that higher capillary density in L4 would tend to increase L4 tissue pO2. Higher capillary/tissue pO2, however, should not bias the ODACITI fit that relies on the periarteriolar gradient. Rather, higher capillary/tissue pO2 would be seen by ODACITI as the hidden variable R0 moving closer to the center arteriole – see Major Comment #1 of Reviewer 2.

*The impact of the paper would be improved if they measured the tissue O2 change to a sensory stimulation. Although, this may be beyond the scope of the current study.*

We agree with the Reviewer that measuring CMRO2 change in response to stimulation is important. While a stimulation/task study is currently underway in our laboratory, the results at this point are too preliminary to be included in the current manuscript. Our preference would be to publish these results in due time as a follow up paper.

**Reviewer #4**

*This manuscript uses a model that was recently developed and published to quantify the oxygen metabolic rate in absolute units (u-mol/ml/min) in the top 4 layers of mouse cortex (L1, L2/3, L4) using a red-shifted exogenous phosphorescent oxygen sensor and two-photon fluorescence lifetime imaging. The authors report summary PO2 levels from near a feeding artery (upper 35% tail) and the tissue PO2 levels from tissue in these locations (lower 35% tail). These "raw" data show that tissue PO2 in L4 is highest and L1 is lowest despite arterial PO2 being roughly the same between these layers. As such, the model developed (based on the traditional and well established Krogh model) estimates that tissue PO2 in L4 is lowest, which is unexpected given the high amount of cytochrome-c present in L4 neurons. The work is fairly clear, the simulation helps setup and explain the model, before the awake head-fixed in vivo results are presented. Model selection is well justified (oxygen gradients around arteries provide direct information of tissue CMRO2). Potential weaknesses in the model selected are also reasonably explained.*

*These findings are unexpected; however, the L1 CMRO2 estimates are considerably variable. It appears about 3 lines trend down in Fig 3C and 2 remain fairly flat. If this is indeed the case, this should be more clearly presented. The authors do use a reasonable statistical model to use all the data to test for significance. However, the results come from 4 (or 5) mice, a relatively low number, although much of their findings does support their claim. If possible, adding one more mouse would be helpful, and/or at least adding a sentence in the results (and may be discussion) to acknowledge L1 variability would be appropriate. Adding a panel in Figure 1 or in the Supplementary Material with the average PO2 vs. depth for each mouse (akin to Fig 3C) would be very valuable also. This is fantastic data from intricate and complex experiments.*

We thank the Reviewer for appreciating the value of our data and complexity of experimental procedures. We have added 4 more mice to increase the overall sample size to 8 animals, 11 arterioles, 51 measurement planes. We also added a new **Supplementary Figure 3** that shows the average pO2 as a function of depth for each mouse that we append below.

**Supplementary Figure 3. Average laminar profile of pO_2_**

Mean pO_2_ calculated by averaging all points measured at the same cortical depth plotted as a function of depth; each line corresponds to a set of measurements acquired along one diving arteriole. Subjects are color-coded; in three cases two arterioles were measured per subject.

Note that these values do not represent the mean tissue pO_2_ because they are biased towards highly oxygenated periarteriolar regions (all measurement grids in this study were centered around penetrating arterioles).

*It would be helpful to discuss how CMRO2 estimated from the neighborhood of arteries could be compared with tissue CMRO2 estimates obtained using the layer tissue PO2 measurements. For example, the authors should discuss how likely is the following rationale. If the venous PO2 is similar along any ascending vein and CBF is relatively uniform, how can these values be explained? Would this instead suggest that venous PO2 varies between layers? Venous PO2 could be estimated using a similar approach to the arterial gradient. Would heterogenous flow along capillary networks explain the difference in CMRO2 (the variability of tissue PO2 between layers does not appear that different -- error bar in figure 1). These scenarios might also help discuss the variability of the results presented.*

We agree with the Reviewer that we could estimate layer-specific CMRO2 if we had information about layer-specific CBF and intravascular O2 saturation of the feeding arterioles and draining veins (SaO2 and SvO2) at each layer using the following equation: CMRO2=CBF(SaO2-SvO2)=SaO2xOEF. We recently published a study where we measured the baseline *intravascular* pO2 in awake mice [26]. From this study we know that there is an increase in venous pO2/SO2 with decreasing depth, most likely due to short capillary paths in upper layers effectively shunting highly oxygenated blood from artery to vein:

In the same study we also showed that speed of capillary RBC flow is similar across layers. Given higher capillary density in L4, this implies that L4 has higher baseline CBF. So, neither SvO2 nor CBF can be assumed to be uniform along the vertical axis. As a result of this complexities, we were unable to compare CMRO2 across cortical layers based on the intravascular pO2 measurements only. These results also motivated the current study as a more feasible approach for estimation of layer-specific CMRO2.

Regarding heterogeneity in capillary flow potentially explaining the difference in CMRO2, in our view CMRO2 is governed by the tissue metabolic needs and should not be affected by vascular organization (in healthy brain) as long as there is sufficient tissue pO2 to sustain mitochondrial ATP production. We know from studies by David Wilson who quantified ATP production as a function of pO_2_ [20] that the range of tissue pO_2_ values reported here as well as in previous studies in healthy adult mice [21, 22] should be more than sufficient.

*Other comments are minor. Please use umol not um when placing units in the figures as this is easily confused with micro-meters.*

Done.

*If you have estimates for the PSF dimension for imaging and FLIM, please provide them to understand the sampling FLIM volume vs. regular imaging.*

Strictly speaking, estimation of PSF would require sub-micron phosphorescence targets (beads) that would be below the diffraction limit of the imaging system (determined by the objective NA and the wavelength). Because we are using water-immersion objectives and our pO2 probes are water-soluble, we would have to embed the probe in some polymer such as PMMA. We have not done it yet. Also, we cannot use fluorescent targets of the same wavelength, because phosphorescence PSF depends on the degree of saturation of the excited state [38]. As of now, we estimate the upper limit of our PSF in the following experiment. Our phantom consists of Oxyphor 2P solution squeezed in between two glass coverslips. Using Optical Coherence Tomography (OCT), we determined that the gap in between the glass surfaces was 2 microns. Then, we aligned the glass surface with the horizontal (table) plane and used 2PLM to step through the phantom by moving the objective in z (the optical path axis) in steps of 1 micron. The resulting phosphorescence intensity is shown in panel (A) below. The full width at the half max (FWHM) of this profile is ~14 microns. By subtracting the 2-micron thickness of the phantom, we arrive as ~12-micron FWHM in z.

For xy, we located two air bubbles separated by ~10 microns of the dye solution within the phantom and placed a line crossing the solution-filled gap between the bubbles. Panel (B) below shows the phosphorescence intensity profile measured along this line. The rising edge estimated as 10% to 90% from the max was ~1.5 microns.

These measurements help set an upper (the worst possible) boundary for our resolution. Because neither of these measurements can be treated as PSF quantification, we prefer not to include these data in the manuscript.

**We thank again all Reviewers for their comments that helped us improve the quality of our manuscript.**

**References**

1.	Buxton RB. Interpreting oxygenation-based neuroimaging signals: the importance and the challenge of understanding brain oxygen metabolism. Front Neuroenergetics. 2010;2:8. Epub 2010/07/10. doi: 10.3389/fnene.2010.00008. PubMed PMID: 20616882; PubMed Central PMCID: PMCPMC2899519.

2.	Gagnon L, Sakadzic S, Lesage F, Musacchia JJ, Lefebvre J, Fang Q, et al. Quantifying the microvascular origin of BOLD-fMRI from first principles with two-photon microscopy and an oxygen-sensitive nanoprobe. J Neurosci. 2015;35(8):3663-75. doi: 10.1523/JNEUROSCI.3555-14.2015. PubMed PMID: 25716864; PubMed Central PMCID: PMCPMC4339366.

3.	Uhlirova H, Kilic K, Tian P, Sakadzic S, Gagnon L, Thunemann M, et al. The roadmap for estimation of cell-type-specific neuronal activity from non-invasive measurements. Philos Trans R Soc Lond B Biol Sci. 2016;371(1705). doi: 10.1098/rstb.2015.0356. PubMed PMID: 27574309; PubMed Central PMCID: PMCPMC5003857.

4.	Griffeth VE, Perthen JE, Buxton RB. Prospects for quantitative fMRI: investigating the effects of caffeine on baseline oxygen metabolism and the response to a visual stimulus in humans. Neuroimage. 2011;57(3):809-16. Epub 2011/05/19. doi: 10.1016/j.neuroimage.2011.04.064. PubMed PMID: 21586328; PubMed Central PMCID: PMCPMC3172084.

5.	Perthen JE, Lansing AE, Liau J, Liu TT, Buxton RB. Caffeine-induced uncoupling of cerebral blood flow and oxygen metabolism: a calibrated BOLD fMRI study. Neuroimage. 2008;40(1):237-47. Epub 2008/01/15. doi: 10.1016/j.neuroimage.2007.10.049. PubMed PMID: 18191583; PubMed Central PMCID: PMCPMC2716699.

6.	Polimeni JR, Wald LL. Magnetic Resonance Imaging technology-bridging the gap between noninvasive human imaging and optical microscopy. Curr Opin Neurobiol. 2018;50:250-60. Epub 2018/05/14. doi: 10.1016/j.conb.2018.04.026. PubMed PMID: 29753942; PubMed Central PMCID: PMCPMC6015259.

7.	Ugurbil K. Imaging at ultrahigh magnetic fields: History, challenges, and solutions. Neuroimage. 2018;168:7-32. Epub 2017/07/13. doi: 10.1016/j.neuroimage.2017.07.007. PubMed PMID: 28698108; PubMed Central PMCID: PMCPMC5758441.

8.	Yu X, Qian C, Chen DY, Dodd SJ, Koretsky AP. Deciphering laminar-specific neural inputs with line-scanning fMRI. Nat Methods. 2014;11(1):55-8. Epub 2013/11/19. doi: 10.1038/nmeth.2730. PubMed PMID: 24240320; PubMed Central PMCID: PMCPMC4276040.

9.	Wald LL. The future of acquisition speed, coverage, sensitivity, and resolution. Neuroimage. 2012;62(2):1221-9. Epub 2012/03/17. doi: 10.1016/j.neuroimage.2012.02.077. PubMed PMID: 22421052; PubMed Central PMCID: PMCPMC3409932.

10.	Polimeni JR, Fischl B, Greve DN, Wald LL. Laminar analysis of 7T BOLD using an imposed spatial activation pattern in human V1. Neuroimage. 2010;52(4):1334-46. Epub 2010/05/13. doi: 10.1016/j.neuroimage.2010.05.005. PubMed PMID: 20460157; PubMed Central PMCID: PMCPMC3130346.

11.	Goense J, Bohraus Y, Logothetis NK. fMRI at High Spatial Resolution: Implications for BOLD-Models. Front Comput Neurosci. 2016;10:66. Epub 2016/07/23. doi: 10.3389/fncom.2016.00066. PubMed PMID: 27445782; PubMed Central PMCID: PMCPMC4923185.

12.	Turner R. Uses, misuses, new uses and fundamental limitations of magnetic resonance imaging in cognitive science. Philos Trans R Soc Lond B Biol Sci. 2016;371(1705). Epub 2016/08/31. doi: 10.1098/rstb.2015.0349. PubMed PMID: 27574303; PubMed Central PMCID: PMCPMC5003851.

13.	Dumoulin SO, Fracasso A, van der Zwaag W, Siero JCW, Petridou N. Ultra-high field MRI: Advancing systems neuroscience towards mesoscopic human brain function. Neuroimage. 2018;168:345-57. Epub 2017/01/18. doi: 10.1016/j.neuroimage.2017.01.028. PubMed PMID: 28093360.

14.	Hirano Y, Stefanovic B, Silva AC. Spatiotemporal evolution of the functional magnetic resonance imaging response to ultrashort stimuli. J Neurosci. 2011;31(4):1440-7. Epub 2011/01/29. doi: 10.1523/JNEUROSCI.3986-10.2011. PubMed PMID: 21273428; PubMed Central PMCID: PMCPMC3078723.

15.	Siero JC, Hendrikse J, Hoogduin H, Petridou N, Luijten P, Donahue MJ. Cortical depth dependence of the BOLD initial dip and poststimulus undershoot in human visual cortex at 7 Tesla. Magn Reson Med. 2015;73(6):2283-95. Epub 2014/07/06. doi: 10.1002/mrm.25349. PubMed PMID: 24989338; PubMed Central PMCID: PMCPMC4282631.

16.	Goense J, Merkle H, Logothetis NK. High-resolution fMRI reveals laminar differences in neurovascular coupling between positive and negative BOLD responses. Neuron. 2012;76(3):629-39. Epub 2012/11/13. doi: 10.1016/j.neuron.2012.09.019. PubMed PMID: 23141073; PubMed Central PMCID: PMCPMC5234326.

17.	Woolsey TA, Rovainen CM, Cox SB, Henegar MH, Liang GE, Liu D, et al. Neuronal units linked to microvascular modules in cerebral cortex: response elements for imaging the brain. Cerebral cortex. 1996;6(5):647-60. Epub 1996/09/01. doi: 10.1093/cercor/6.5.647. PubMed PMID: 8921201.

18.	Blinder P, Tsai PS, Kaufhold JP, Knutsen PM, Suhl H, Kleinfeld D. The cortical angiome: an interconnected vascular network with noncolumnar patterns of blood flow. Nat Neurosci. 2013;16(7):889-97. Epub 2013/06/12. doi: 10.1038/nn.3426. PubMed PMID: 23749145; PubMed Central PMCID: PMCPMC4141079.

19.	Adams DL, Piserchia V, Economides JR, Horton JC. Vascular Supply of the Cerebral Cortex is Specialized for Cell Layers but Not Columns. Cerebral cortex. 2015;25(10):3673-81. doi: 10.1093/cercor/bhu221. PubMed PMID: WOS:000366454000033.

20.	Wilson DF, Harrison DK, Vinogradov A. Mitochondrial cytochrome c oxidase and control of energy metabolism: measurements in suspensions of isolated mitochondria. J Appl Physiol (1985). 2014;117(12):1424-30. Epub 2014/10/18. doi: 10.1152/japplphysiol.00736.2014. PubMed PMID: 25324517.

21.	Lyons DG, Parpaleix A, Roche M, Charpak S. Mapping oxygen concentration in the awake mouse brain. Elife. 2016;5. Epub 2016/02/03. doi: 10.7554/eLife.12024. PubMed PMID: 26836304; PubMed Central PMCID: PMCPMC4775210.

22.	Lu X, Moeini M, Li B, de Montgolfier O, Lu Y, Belanger S, et al. Voluntary exercise increases brain tissue oxygenation and spatially homogenizes oxygen delivery in a mouse model of Alzheimer's disease. Neurobiol Aging. 2020;88:11-23. Epub 2019/12/24. doi: 10.1016/j.neurobiolaging.2019.11.015. PubMed PMID: 31866158.

23.	Tsai PS, Kaufhold JP, Blinder P, Friedman B, Drew PJ, Karten HJ, et al. Correlations of neuronal and microvascular densities in murine cortex revealed by direct counting and colocalization of nuclei and vessels. J Neurosci. 2009;29(46):14553-70. Epub 2009/11/20. doi: 10.1523/JNEUROSCI.3287-09.2009. PubMed PMID: 19923289; PubMed Central PMCID: PMCPMC4972024.

24.	Jiang X, Wang G, Lee AJ, Stornetta RL, Zhu JJ. The organization of two new cortical interneuronal circuits. Nat Neurosci. 2013;16(2):210-8. doi: 10.1038/nn.3305. PubMed PMID: 23313910; PubMed Central PMCID: PMCPMC3589105.

25.	Wang DB, Blocher NC, Spence ME, Rovainen CM, Woolsey TA. Development and remodeling of cerebral blood vessels and their flow in postnatal mice observed with in vivo videomicroscopy. J Cereb Blood Flow Metab. 1992;12(6):935-46. Epub 1992/11/01. doi: 10.1038/jcbfm.1992.130. PubMed PMID: 1400647.

26.	Li B, Esipova TV, Sencan I, Kilic K, Fu B, Desjardins M, et al. More homogeneous capillary flow and oxygenation in deeper cortical layers correlate with increased oxygen extraction. Elife. 2019;8. Epub 2019/07/16. doi: 10.7554/eLife.42299. PubMed PMID: 31305237; PubMed Central PMCID: PMCPMC6636997.

27.	Markram H, Toledo-Rodriguez M, Wang Y, Gupta A, Silberberg G, Wu C. Interneurons of the neocortical inhibitory system. Nat Rev Neurosci. 2004;5(10):793-807. doi: 10.1038/nrn1519. PubMed PMID: 15378039.

28.	Santuy A, Turegano-Lopez M, Rodriguez JR, Alonso-Nanclares L, DeFelipe J, Merchan-Perez A. A Quantitative Study on the Distribution of Mitochondria in the Neuropil of the Juvenile Rat Somatosensory Cortex. Cerebral cortex. 2018;28(10):3673-84. Epub 2018/07/31. doi: 10.1093/cercor/bhy159. PubMed PMID: 30060007; PubMed Central PMCID: PMCPMC6132283.

29.	Sakadzic S, Yaseen MA, Jaswal R, Roussakis E, Dale AM, Buxton RB, et al. Two-photon microscopy measurement of cerebral metabolic rate of oxygen using periarteriolar oxygen concentration gradients. Neurophotonics. 2016;3(4):045005. doi: 10.1117/1.NPh.3.4.045005. PubMed PMID: 27774493; PubMed Central PMCID: PMCPMC5066455.

30.	Krogh A. The number and distribution of capillaries in muscles with calculations of the oxygen pressure head necessary for supplying the tissue. J Physiol. 1919;52(6):409-15. Epub 1919/05/20. doi: 10.1113/jphysiol.1919.sp001839. PubMed PMID: 16993405; PubMed Central PMCID: PMCPMC1402716.

31.	Ayata C, Shin HK, Salomone S, Ozdemir-Gursoy Y, Boas DA, Dunn AK, et al. Pronounced hypoperfusion during spreading depression in mouse cortex. J Cereb Blood Flow Metab. 2004;24(10):1172-82. Epub 2004/11/06. doi: 10.1097/01.WCB.0000137057.92786.F3. PubMed PMID: 15529018.

32.	Yuzawa I, Sakadzic S, Srinivasan VJ, Shin HK, Eikermann-Haerter K, Boas DA, et al. Cortical spreading depression impairs oxygen delivery and metabolism in mice. J Cereb Blood Flow Metab. 2012;32(2):376-86. Epub 2011/10/20. doi: 10.1038/jcbfm.2011.148. PubMed PMID: 22008729; PubMed Central PMCID: PMCPMC3272607.

33.	Sencan I, Esipova T, Kilic K, Li B, Desjardins M, Yaseen MA, et al. Optical measurement of microvascular oxygenation and blood flow responses in awake mouse cortex during functional activation. J Cereb Blood Flow Metab. 2020:271678X20928011. Epub 2020/06/10. doi: 10.1177/0271678X20928011. PubMed PMID: 32515672.

34.	Theer P, Denk W. On the fundamental imaging-depth limit in two-photon microscopy. J Opt Soc Am A Opt Image Sci Vis. 2006;23(12):3139-49. PubMed PMID: 17106469.

35.	Rupprecht P, Prevedel R, Groessl F, Haubensak WE, Vaziri A. Optimizing and extending light-sculpting microscopy for fast functional imaging in neuroscience. Biomed Opt Express. 2015;6(2):353-68. Epub 2015/03/18. doi: 10.1364/BOE.6.000353. PubMed PMID: 25780729; PubMed Central PMCID: PMCPMC4354592.

36.	Cheng X, Sadegh S, Zilpelwar S, Devor A, Tian L, Boas DA. Comparing the fundamental imaging depth limit of two-photon, three-photon, and non-degenerate two-photon microscopy. Opt Lett. 2020;45(10):2934-7. Epub 2020/05/16. doi: 10.1364/OL.392724. PubMed PMID: 32412504; PubMed Central PMCID: PMCPMC8059139.

37.	Sadegh S, Yang MH, Ferri CGL, Thunemann M, Saisan PA, Wei Z, et al. Efficient non-degenerate two-photon excitation for fluorescence microscopy. Opt Express. 2019;27(20):28022-35. Epub 2019/11/07. doi: 10.1364/OE.27.028022. PubMed PMID: 31684560; PubMed Central PMCID: PMCPMC6825618.

38.	Sinks L, Roussakis E, Sakadžic S, Robbins G, Hammer D, Devor A, et al. Two-photon phosphorescence lifetime microscopy (2PLM) for high resolution imaging of oxygen: SPIE; 2011.
